# Loss-of-Function Mutations in the Penicillin-Binding Protein PonA1 Confer Agar-Dependent Resistance to Durlobactam in *Mycobacterium abscessus*

**DOI:** 10.3390/antibiotics15010007

**Published:** 2025-12-20

**Authors:** Dereje Abate Negatu, Wassihun Wedajo Aragaw, Min Xie, Véronique Dartois, Thomas Dick

**Affiliations:** 1 Center for Discovery and Innovation, Hackensack Meridian Health, 111 Ideation Way, Nutley, NJ 07110, USA; dereje.negatu@sluhn.org (D.A.N.); wassihunwedajo.aragaw@hmh-cdi.org (W.W.A.); min.xie@hmh-cdi.org (M.X.); thomas.dick@hmh-cdi.org (T.D.); 2 Department of Medical Sciences, Hackensack Meridian School of Medicine, 123 Metro Boulevard, Nutley, NJ 07110, USA; 3 Department of Microbiology and Immunology, Georgetown University, Washington, DC 20057, USA

**Keywords:** *Mycobacterium abscessus*, durlobactam, acquired resistance

## Abstract

**Background**: Infections caused by the multidrug-resistant pathogen *Mycobacterium abscessus* (*Mab*) are notoriously difficult to treat. The novel β-lactamase inhibitor durlobactam, in combination with β-lactams, shows potent bactericidal activity against *Mab*, but the potential for acquired resistance remains a clinical concern. **Objectives**: To identify and characterize mechanisms of acquired resistance to durlobactam in *Mab*. **Methods**: In vitro single-step resistance selection was performed by plating wild-type *Mab* ATCC 19977 and by transcriptional silencing using a CRISPR interference (CRISPRi) system. Minimum inhibitory concentrations (MICs) were determined by both an agar-based method and broth microdilution. **Results**: Whole-genome sequencing of durlobactam-resistant mutants identified loss-of-function mutations in *ponA1*, a gene encoding a class A penicillin-binding protein involved in cell wall synthesis. Targeted deletion of *ponA1* (Δ*ponA1*) and CRISPRi-mediated knockdown of *ponA1* expression both recapitulated the resistance phenotype, resulting in a significant increase in the durlobactam MIC on solid agar media. Strikingly, broth microdilution MICs remained largely unaffected. **Conclusions**: Inactivation of the peptidoglycan synthase PonA1 is a novel mechanism of resistance to durlobactam in *Mab* that is phenotypically expressed only during growth on solid surfaces. This finding identifies a specific genetic pathway for resistance and highlights that standard broth-based susceptibility testing could miss clinically relevant resistance mechanisms.

## 1. Introduction

*Mycobacterium abscessus* (*Mab*) is an emerging opportunistic pathogen responsible for chronic and debilitating pulmonary infections, particularly in individuals with underlying lung conditions [1,2]. Treatment of *Mab* is exceptionally challenging due to its extensive intrinsic and acquired resistance to most available antibiotics [3], leading to poor clinical outcomes and cure rates often below 50% [4]. This clinical reality underscores the urgent need for novel treatment strategies.

The revitalization of β-lactam antibiotics through combination with potent β-lactamase inhibitors (BLIs) represents a promising therapeutic avenue. Durlobactam (DUR), a next-generation diazabicyclooctane (DBO) inhibitor [5,6], has demonstrated remarkable potency against *Mab*. Its efficacy against mycobacteria stems from a dual mechanism of action: it potently inactivates the major β-lactamase, BlaC or Bla_Mab_, while also exhibiting intrinsic antibacterial activity through the direct inhibition of cell wall synthesis enzymes, including penicillin-binding proteins (PBPs) and L,D-transpeptidases [7,8]. Against *Mab*, this multi-target engagement results in profound synergy with partner β-lactams like imipenem and ceftriaxone, achieving potent bactericidal activity at clinically relevant concentrations [9,10,11,12].

The long-term clinical success of any new antimicrobial agent is contingent upon understanding and anticipating the pathogen’s evolutionary pathways to resistance. While several mechanisms that compromise β-lactam-based therapies in *Mab* have been described—including upregulation of Bla_Mab_, reduced cell wall permeability via porin loss, and induction of drug tolerance through mutations in the stress-response regulator RshA [11,13,14]—mechanisms conferring direct resistance to advanced DBO inhibitors like DUR are not well understood. Since target-based mutations are considered uncommon for β-lactams in *Mab* due to the functional redundancy of peptidoglycan synthesis enzymes, it is critical to prospectively identify the most likely pathways to resistance.

In this study, we sought to identify and validate novel mechanisms of acquired resistance to DUR in *Mab*. Through in vitro single-step selection experiments, we discovered that high level resistance consistently arises through mutations in *ponA1*, encoding a class A PBP that performs both transglycosylase and D,D-transpeptidase activities. We confirmed this unexpected finding by demonstrating that both targeted deletion and CRISPRi-mediated knockdown of *ponA1* are sufficient to confer DUR resistance.

## 2. Results

**Figure 1:**
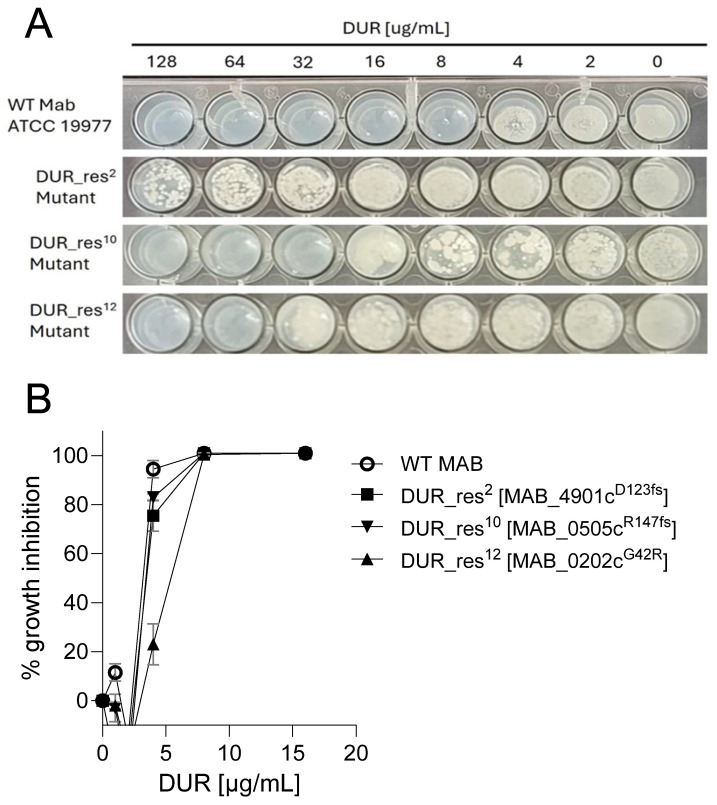
DUR-resistant *Mab* mutants display moderate or high-level resistance on solid medium but weak resistance in broth. (**A**) Agar-based minimum inhibitory concentration (MIC). Cultures of wild-type (WT) *Mab* ATCC 19977 and representative resistant mutants were adjusted to OD_600_ of 0.05, and 15 µL was spotted onto 7H10 agar containing serial dilutions of DUR in 48-well plates. The image shows bacterial growth after 5 days of incubation. (**B**) Dose–response curves of DUR against wild-type and resistant strains in 7H9 broth. Percent growth inhibition was calculated relative to untreated control wells after 3 days of incubation. Mean ± standard deviation (error bars) values from three biological replicates are shown.

To identify genes involved in acquired DUR resistance, we selected spontaneous resistant mutants on 7H10 agar containing 4× the agar MIC of 8 mg/L. We isolated fourteen mutants in two independent rounds of selection, seven of which showed high level resistance, with an agar MIC > 128 mg/L. The remainder displayed moderate resistance with agar MICs ranging from 32 to 64 mg/L (Table 1, Figure 1A). The frequency of resistance was ~10^−7^/CFU. Interestingly, all fourteen mutants showed a weak (twofold) increase in broth MIC compared to the wild-type strain (Figure 1B).

Whole-genome sequencing revealed that high-level resistant mutants all carried mutations in MAB_4901c encoding the bifunctional transglycosylase/transpeptidase PonA1 involved in peptidoglycan synthesis [15]: two frameshift, three nonsense, one in-frame deletion and one missense mutation (Table 1). The nature of the mutations in *ponA1* suggested that loss-of-function causes the resistance phenotype. To confirm this hypothesis, we generated a complete deletion of MAB_4901c via allelic exchange, verified by whole genome sequencing (Figure S1). The MIC pattern of the *ponA1* knockout strain was identical to the original spontaneous frameshift mutant strain *Mab* DUR_res^2^ with high-level (>128 mg/L) resistance on solid medium (Table 2).

Mutations conferring moderate-level resistance were found in either MAB_0505c or MAB_0205c, whose functions are currently unknown (Table 1). From there on, we focused on characterizing the PonA1-mediated mechanism of high-level resistance to DUR.

**Figure 2:**
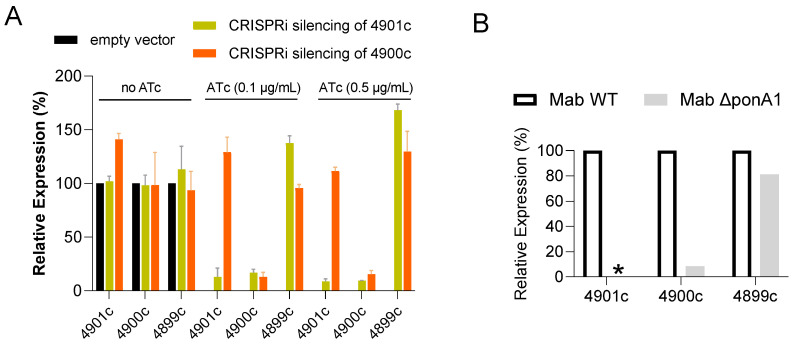
Quantification of *ponA1* and downstream mRNA transcripts following CRISPRi-mediated gene knockdown and knockout. (**A**) Relative expression of MAB_4901c (*ponA1*), MAB_4900c (hypothetical protein, separated by 4 bp from *ponA1*), and MAB_4899c (*rpsF*; 30S subunit ribosomal protein S6, separated by ~170 bp from MAB_4900c) in the CRISPRi knockdown strains without and with inducer ATc. (**B**) Relative expression of MAB_4901c, MAB_4900C and MAB_4899c in ∆*ponA1* compared to parental wild-type strain. *: no PCR product detected. Expression levels were normalized to *sigA* mRNA and are shown relative to the corresponding control strain. Details of strain engineering are provided in Figures S1 and S2.

Since genomic analysis suggests that *ponA1* (MAB_4901c) and the downstream gene MAB_4900c, encoding a hypothetical protein, form a putative operon, we hypothesized that the mutations in *ponA1* might exert a polar effect on the expression of MAB_4900c. Using the CRISPRi system for gene expression knockdown, we silenced either *ponA1* or MAB_4900c in two separate engineered strains (Figure S2) and quantified their respective mRNA levels by qRT-PCR. As expected, induction of *ponA1* silencing with 0.5 mg/L anhydro-tetracycline led to >85% reduction in both *ponA1* and MAB_4900c mRNA, while targeting MAB_4900c only suppressed the expression of MAB_4900c, confirming the operon structure (Figure 2A). The polar effect of *ponA1* on the transcription of MAB_4900c was recapitulated in the ∆*ponA1* knockout strain (Figure 2B). Next, we evaluated the effect of targeted gene silencing on DUR resistance. Silencing of *ponA1* resulted in a 4-fold increase in DUR agar MIC, phenocopying the resistance observed in the spontaneous mutants and the ∆*ponA1* deletion strain. However, specific silencing of MAB_4900c alone had no effect on DUR susceptibility, indicating that loss of *ponA1* function is the primary driver of resistance. The broth MICs of both knockdown strains remained unchanged.

## 3. Discussion

In this study, we identify a novel mechanism of acquired resistance to DUR in *Mab*. Through single-step in vitro selection, we consistently isolated mutants with alterations in *ponA1*, encoding the class A PBP PonA1 [16]. This finding is notable because resistance to β-lactam-based therapies in *Mab* has predominantly been associated with mechanisms that protect the β-lactam or induce a general state of drug tolerance [13,14]. In *M. tuberculosis*, biochemical studies showed that PonA1 is one of the targets of DUR [8]. Thus, inactivation of PonA1 represents a direct, target-based resistance mechanism, a pathway previously thought to be uncommon for β-lactams in this pathogen and in mycobacteria in general due to the redundancy of β-lactam targets [17,18].

The observation that inactivating a drug’s target confers resistance is counterintuitive. Here, this could be explained by DUR’s multi-target mechanism of action and redundancy in peptidoglycan synthesis. It is plausible that while DUR inhibits several PBPs and L,D-transpeptidases, binding to PonA1 is a primary driver of its antimicrobial activity by creating lethal imbalances in peptidoglycan synthesis. A complete removal of this key target via a loss-of-function would allow *Mab* to overcome the lethal effect of corruption of cell wall synthesis. The viability of *ponA1* loss-of-function mutants strongly suggests that other PBPs can compensate for its loss. This functional redundancy is a known feature of the mycobacterial cell wall synthesis machinery [18]. Under the selective pressure of DUR, *Mab* can sacrifice the PonA1-mediated pathway—most vulnerable to the drug—and rely on alternative, less-susceptible pathways for survival.

This mechanism mirrors findings in *M. tuberculosis*, where deletion of the homologous PBP PonA2 confers resistance to cephalosporins [19]. *M. tuberculosis* evades the specific lethality induced by b-lactam inhibition of PonA2 by sacrificing PonA2 and relying on redundant transpeptidation pathways, specifically those mediated by L,D-transpeptidases [19].

A striking and critical feature of this resistance mechanism in *Mab* is its dependence on culture conditions. While *ponA1* mutants exhibit a pronounced increase in MIC on agar, they appeared almost fully susceptible when tested by standard broth microdilution. This disconnect between agar- and broth-based susceptibility suggests that the resistance phenotype is linked to a physiological state specific to surface-based growth. Two non-mutually exclusive hypotheses could explain this phenomenon. First, the loss of PonA1 may alter cell wall architecture and colony properties in a way that limits drug diffusion through the nascent “biofilm” on a solid agar surface [20], a barrier that would not exist in planktonic cells in a well-mixed broth. Second, growth on a solid surface may induce a specific metabolic or stress state in which the loss of PonA1 is less detrimental, or in which compensatory pathways are activated, allowing the resistance phenotype to manifest.

These findings have potential clinical implications. Standard antimicrobial susceptibility testing is typically performed using broth-based methods [21]. Our results demonstrate that such methods would fail to detect this resistance mechanism, potentially leading to the misclassification of a resistant isolate as susceptible and subsequent therapeutic failure. The possible clinical relevance of this agar-dependent resistance is underscored by the fact that mycobacterial growth in the lungs often occurs in biofilm-like aggregates or microcolonies [22], a state that is more closely modeled by growth on a solid surface than in liquid culture [23].

## 4. Materials and Methods

*Mab* subsp. *abscessus* ATCC 19977 was used as the wild-type strain in all experiments. Spontaneous resistant mutants were selected by plating approximately 10^9^ colony-forming units (CFU) of wild-type ATCC 19977 onto 7H10 solid medium containing 4× the agar minimum inhibitory concentration (MIC) of DUR (Cat# HY-117974, MedChemExpress LLC, Monmouth Junction, NJ, USA). Plates were incubated at 37 °C for 5 days. Resistant colonies were re-streaked on selective plates to confirm the resistance phenotype. Two independent selections were performed to ensure the reproducibility of the results. Broth and agar minimum inhibitory concentrations (MICs) were determined as previously described [24].

To knockout MAB_4901c, an allelic exchange substrate (AES) was employed as described in Figure S1. The AES contained a 500 bp region upstream of *ponA1* (USH, upstream homology region) and a region 500 bp downstream of *ponA1* (DSH, downstream homology region), flanking a cassette comprised of an apramycin resistance gene (Apra R), and an mScarlet reporter gene under control of the PLeft* promoter to enable selection and identification of recombinants [25,26]. The synthesized AES was PCR amplified using primers 5′-CGGACCGCCGGTGTGCCGTCGTACTG-3′ and 5′-CTGGTTAGCGTGCGATTGCAGAGAC-3′, electroporated into *Mab* ATCC 19977 and plated on 7H10 agar containing 50 mg/L apramycin. After 7 days of incubation at 37 °C, colonies were screened visually for red color (mScarlet expression). MAB_4901c knockout was confirmed by whole-genome sequencing.

A CRISPR interference (CRISPRi)-dCas9 system provided on the pLJR962 plasmid [27] was utilized to knock down gene expression, as previously described [28]. Single-guide RNAs (sgRNAs) targeting the N-terminal coding region of each gene were designed based on predicted strength using the sgRNA Design Tool version 2.0 (https://pebble.rockefeller.edu/tools/sgrna-design (accessed on 28 January 2025). The sgRNA target sequences and protospacer adjacent motif (PAM) sequences are provided in Figure S2. Complementary oligos for each sgRNA were synthesized by Azenta Life Sciences, South Plainfield, NJ, USA. To construct the sgRNA expression plasmids, the recipient vector pLJR962 was digested with *Bsm*BI (Thermo Fisher Scientific, Waltham, MA, USA, Cat. No. ER0451). The complementary top and bottom oligos for each target were annealed in a thermocycler by incubating at 95 °C for 2 min, followed by a gradual ramp-down to 25 °C at a rate of −0.1 °C per second. The resulting annealed duplexes were ligated into the *Bsm*BI-digested pLJR962 vector using T4 DNA Ligase overnight at 16 °C. Correct insertion of the sgRNA cassette was verified by Sanger sequencing. Gene expression knockdown was induced on solid and in liquid medium with anhydrotetracycline (ATc) as described [28].

For total RNA extraction, frozen pellets flash-frozen in 1 mL of TRIzol Reagent (Ambion, Austin, TX, USA) in 2 mL Lysing Matrix B tubes (MP Biomedicals, Irvine, CA, USA), were thawed and subjected to bead beating twice for 30 s at maximum speed using a Fisherbrand Bead Mill 24 Homogenizer. RNA was purified using the RNeasy Mini kit (Qiagen, Venlo, The Netherlands) with on-column DNase I treatment (Qiagen). First-strand cDNA synthesis was performed using random hexamers and SuperScript IV Reverse Transcriptase (Invitrogen, Carlsbad, CA, USA). Following qRT-PCR using primers listed in Table S1, gene expression levels were normalized to the housekeeping gene *sigA* (Mab_3009), and relative expression differences were calculated using the 2−ΔΔCt method [29].

## 5. Conclusions

Our study demonstrates that *Mab* can develop resistance to DUR through loss-of-function mutations in *ponA1*, suggesting resistance driven by the evasion of a drug-induced toxic gain of function, rather than simple target inhibition. We propose that DUR binding to PonA1 does not merely silence the enzyme but actively corrupts cell wall synthesis. By eliminating the target protein PonA1, *Mab* removes the substrate for this lethal malfunctioning, allowing survival via redundant transpeptidation pathways. Crucially, this resistance phenotype is agar-dependent, manifesting mainly during growth on solid media. This discrepancy likely reflects the distinct metabolic or structural constraints of biofilm-like growth on agar, which may buffer the physiological cost of PonA1 loss or enhance the efficacy of alternative synthesis pathways compared to planktonic conditions. The identification of this ‘evasion by deletion’ strategy highlights the complex interplay between drug lethality, target essentiality, and growth phase in mycobacteria, underscoring the potential for standard broth-based susceptibility testing to miss clinically relevant resistance mechanisms.

## Figures and Tables

**Table 1:** Characterization of DUR-resistant *Mycobacterium abscessus* mutants.

Strain *	Round of Selection	Agar MIC ** (mg/L)	Broth MIC ** (mg/L)	Mutations			Gene Function
				Gene	DNA Alteration	Amino Acid Sequence Alteration	
WT		8	4	wt	wt	wt	-
DUR_res^1^	1st	>128	8	MAB_4901c (*ponA1*)	1312delC	Q438fs	Penicillin-binding protein
**DUR_res^2^**	1st	>128	8		369_370del	D123fs	
DUR_res^3^	1st	>128	8		C1156T	Q386stop	
DUR_res^4^	2nd	>128	8		G2027T	G676V	
DUR_res^5^	2nd	>128	8		C1333T	Q445stop	
DUR_res^6^	2nd	>128	8		C1333T	Q445stop	
DUR_res^7^	2nd	>128	8		776_778del	259_260del	
DUR_res^8^	1st	32	8	MAB_0505c	439dupC	R147fs	Hypothetical protein
DUR_res^9^	1st	64	8		T608C	L203P	
**DUR_res^10^**	2nd	32	8		439dupC	R147fs	
DUR_res^11^	2nd	32	8		71_82del	24_28del	
**DUR_res^12^**	1st	64	8	MAB_0205c	G124C	G42R	Hypothetical protein
DUR_res^13^	1st	64	8		G124C	G42R	
DUR_res^14^	2nd	64	8		G124C	G42R	

* strains highlighted in bold are those that were selected for further characterization. ** MIC determinations were carried out twice independently yielding the same results.

**Table 2:** DUR susceptibility of spontaneous and engineered *M. abscessus* mutants.

Mab Strain	Agar MIC (mg/L)	Broth MIC (mg/L)	Strain Characteristics
Wild-type	8	4	Wild-type ATCC 19977
DUR_res^2^	>128	8	Spontaneous DUR resistant *ponA1* (MAB_4901c) frameshift mutant (Table 1)
∆*ponA1*	>128	4	Engineered *ponA1* deletion mutant (Figure S1)
*ponA1* KD	32	4	CRISPRi ponA1 knockdown (Figure S2)
MAB_4900c KD	8	4	CRISPRi MAB_4900c (hypothetical protein) knockdown (Figure S2)

## Data Availability

The data supporting the findings of this study are available within the article and its Supplementary Materials.

## Supplementary Materials

The following supporting information can be downloaded at: https://www.mdpi.com/article/10.3390/antibiotics15010007/s1, Figure S1: Deletion of *ponA1* (MAB_4901c) strategy; Figure S2: Transcriptional silencing of MAB_4901c (*ponA1*) and MAB_4900c; Table S1: Primers used in rt-qPCR [25,26,27,28].

## Abbreviations

The following abbreviations are used in this manuscript:

DUR	Durlobactam
*Mab*	*Mycobacterium abscessus*
