# Supplementary material for: Loss-of-Function Mutations in the Penicillin-Binding Protein PonA1 Confer Agar-Dependent Resistance to Durlobactam in Mycobacterium abscessus
Source: Antibiotics (Basel). 2025 Dec 20;15(1):7. doi: 10.3390/antibiotics15010007 (PMC12837516; doi:10.3390/antibiotics15010007)
Supplement: Supplementary file 1 [file antibiotics-15-00007-s001.zip › antibiotics-4028339-supplementary.pdf]

Supplemental Material

Loss-of-Function Mutations in the Penicillin-Binding Protein PonA1 Confer Agar-Dependent Resistance to Durlobactam in *Mycobacterium abscessus*

Dereje Abate Negatu [(1)*], Wassihun Wedajo Aragaw [(1)], Min Xie [(1)], Véronique Dartois [(1,2)^] and Thomas Dick [(1,2,3)]

[(1)] Center for Discovery and Innovation, Hackensack Meridian Health, 111 Ideation Way, Nutley, NJ 07110, USA
[(2)] Department of Medical Sciences, Hackensack Meridian School of Medicine, 123 Metro Boulevard, Nutley, NJ 07110, USA
[(3)] Georgetown University, Department of Microbiology and Immunology, Washington DC 20057, USA

* Present address: Graduate Medical Education, St. Luke's University Hospital, Bethlehem, PA, USA

^Corresponding author: veronique.dartois@hmh-cdi.org

Supplemental Figures

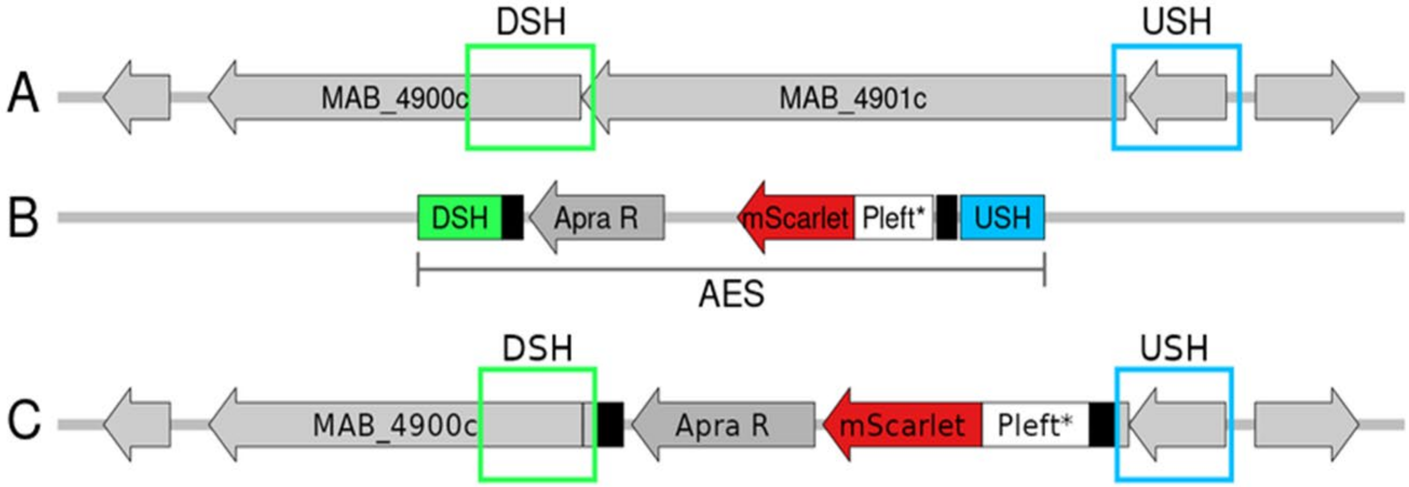

Supplemental Figure S1. Deletion of *ponA1* (MAB_4901c) strategy. (A-B) An allelic exchange substrate (AES) was synthesized by Azenta Life Sciences, South Plainfield, NJ, USA. The AES contained a 500 bp region upstream of *ponA1* (USH, upstream homology region) and a region 500 bp downstream of *ponA1* (DSH, downstream homology region), flanking a cassette comprised of an apramycin resistance gene (Apra R), and an mScarlet reporter gene under control of the PLeft* promoter to enable selection and identification of recombinants [1, 2]. The synthesized AES was PCR amplified using primers 5'-CGGACCGCCGGTGTGCCGTCGTACTG-3' and 5'-CTGGTTAGCGTGCGATTGCAGAGAC-3', electroporated into *Mab* ATCC 19977 and plated on 7H10 agar containing 50 mg/L apramycin. After 7 days of incubation at 37°C, colonies were screened visually for red color (mScarlet expression). **(C)** Whole genome sequencing confirmed the replacement of *ponA1* with the 'Apra R – mScarlet' cassette.

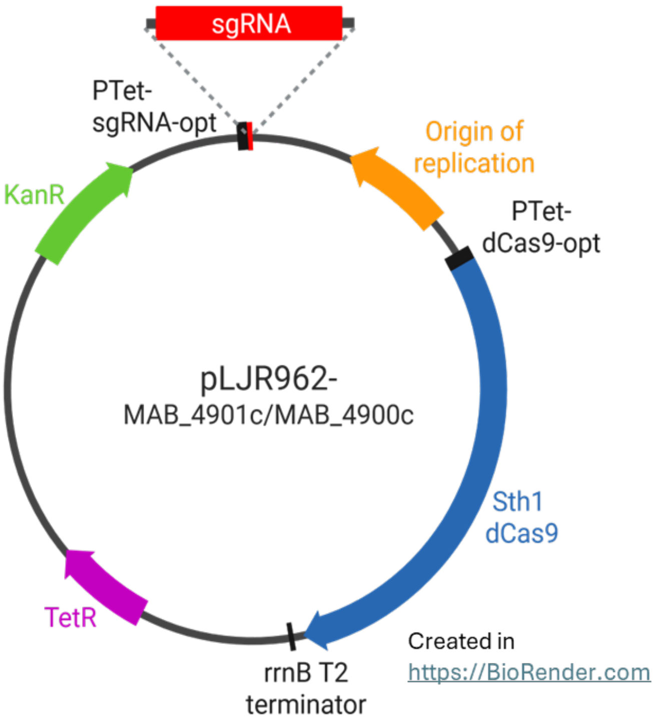

MAB_4901c (*ponA1*)

- sgRNA target = ACGGCGCGCAACAGACCCGAGAA
- PAM seq = CGAGAAG
- Top oligo = GGGAGACGGCGCGCAACAGACCCGAGAA
- Bottom oligo = AAACTTCTCGGGTCTGTTGCGCGCCGTC
- Predicted strength = 0.98

MAB_4900c

- sgRNA target = GCAGGGCGCCTTGCTGGTCCACC
- PAM seq = CCAGAAT
- Top oligo = GGGAGCAGGGCGCCTTGCTGGTCCACC
- Bottom oligo = AAACGGTGGACCAGCAAGGCGCCCTGC
- Predicted strength = 0.99

Supplemental Figure S2. Transcriptional silencing of MAB_4901c (*ponA1*) and MAB_4900c. A CRISPR interference (CRISPRi)-dCas9 system provided on the pLJR962 plasmid [3] was utilized to knock down the expression of the genes, as previously described [4]. Single-guide RNAs (sgRNAs) targeting the N-terminal coding region of each gene were designed based on predicted strength using the sgRNA Design Tool (https://pebble.rockefeller.edu/tools/sgrna-design). The sgRNA target sequences and protospacer adjacent motif (PAM) sequences are provided above. Red font indicate the transcription start site for the sgRNA. Complementary oligos for each sgRNA were synthesized by Azenta Life Sciences, South Plainfield, NJ, USA. To construct the sgRNA expression plasmids, the recipient vector pLJR962 was digested with BsmBI (Thermo Fisher Scientific, Cat. No. ER0451). The complementary top and bottom oligos for each target were annealed in a thermocycler by incubating at 95°C for 2 minutes, followed by a gradual ramp-down to 25°C at a rate of -0.1°C per second. The resulting annealed duplexes were ligated into the BsmBI-digested pLJR962 vector using T4 DNA Ligase overnight at 16°C. PTet-sgRNA-opt, optimized TetR-regulated sgRNA promoter; PTet-dCas9-opt, optimized TetR-regulated dCas9 promoter; KanR, kanamycin resistance gene.

Supplemental Table S1. Primers used in rt-qPCR

Primers	5' - 3'
MAB_4901c_for	CAGGGCATCACCATCAAGAA
MAB_4901c_rev	CTTCAGCATGAGGCGGTAAA
MAB_4900c_for	TCGCAACGGCTTTCAGTAT
MAB_4900c_rev	CTACGCAGGCTGACCAATAG
MAB_4899c_for	CGTCGTCTTGCTTACGAGAT
MAB_4899c_rev	GCAACACGGACTCGTTCA
MAB_3009_for	CCAAGAACCATCTGCTGGAA
MAB_3009_rev	CCAGGTTGCCTTCCTGAAT

References

1. Ganapathy US, Lan T, Krastel P et al. Blocking Bacterial Naphthohydroquinone Oxidation and ADP-Ribosylation Improves Activity of Rifamycins against Mycobacterium abscessus. *Antimicrob Agents Chemother* 2021; **65**: e0097821.
2. Kolbe K, Bell AC, Prosser GA et al. Development and Optimization of Chromosomally-Integrated Fluorescent Mycobacterium tuberculosis Reporter Constructs. *Front Microbiol* 2020; **11**: 591866.
3. Wong AI, Rock JM. CRISPR Interference (CRISPRi) for Targeted Gene Silencing in Mycobacteria. *Methods Mol Biol* 2021; **2314**: 343-64.
4. Kurepina N, Chen L, Composto K et al. CRISPR Inhibition of Essential Peptidoglycan Biosynthesis Genes in Mycobacterium abscessus and Its Impact on beta-Lactam Susceptibility. *Antimicrob Agents Chemother* 2022; **66**: e0009322.
